# Classification of Microarray Data Using Kernel Fuzzy Inference System

**DOI:** 10.1155/2014/769159

**Published:** 2014-08-21

**Authors:** Mukesh Kumar, Santanu Kumar Rath

**Affiliations:** Department of Computer Science and Engineering, NIT Rourkela, Rourkela, Odisha 769008, India

## Abstract

The DNA microarray classification technique has gained more popularity in both research and practice. In real data analysis, such as microarray data, the dataset contains a huge number of insignificant and irrelevant features that tend to lose useful information. Classes with high relevance and feature sets with high significance are generally referred for the selected features, which determine the samples classification into their respective classes. In this paper, kernel fuzzy inference system (K-FIS) algorithm is applied to classify the microarray data (leukemia) using* t*-test as a feature selection method. Kernel functions are used to map original data points into a higher-dimensional (possibly infinite-dimensional) feature space defined by a (usually nonlinear) function *ϕ* through a mathematical process called the* kernel trick*. This paper also presents a comparative study for classification using K-FIS along with support vector machine (SVM) for different set of features (genes). Performance parameters available in the literature such as precision, recall, specificity,* F*-measure, ROC curve, and accuracy are considered to analyze the efficiency of the classification model. From the proposed approach, it is apparent that K-FIS model obtains similar results when compared with SVM model. This is an indication that the proposed approach relies on kernel function.

## 1. Introduction

Accurate diagnosis of the disease, particularly “cancer,” is vital for the successful application of any specific therapy. Even though classification related to cancer diagnosis has been improved over the last decade significantly, still there is a need for its proper diagnosis with less subjective methods. Recent development in diagnosis indicates that DNA microarray provides an insight into cancer classification at the gene level due to their capabilities to measure abundant ribonucleic acid (mRNA) transcripts for thousands of genes concurrently.

Microarray-based gene expression profiling has emerged as an efficient technique for cancer classification as well as for diagnosis, prognosis, and treatment purposes [1–3]. In recent years, DNA microarray technique has shown great impact on determining the informative genes that cause cancer [4, 5].

The major drawback that exists in microarray data is the curse of dimensionality problem; that is, the number of genes *N* far exceeds the number of samples *M* (*N* ≫ *M*), which hinders the useful information of dataset and the computational instability [6]. Therefore, the selection of relevant genes remains a challenge in the analysis of microarray data [1]. The aim of gene selection is to select a small subset of genes from a larger pool, yielding not only good performance of classification but also biologically meaningful insights. Gene selection methods are classified into three types: (a) filter methods, (b) wrapper methods, and (c) embedded methods. Filter methods evaluate a gene subset by looking at the intrinsic characteristics of data with respect to class labels [1], while wrapper methods evaluate the goodness of a gene subset by the accuracy of its learning or classification. Embedded methods are generally referred to algorithms where gene selection is embedded in the construction of the classifier [7].

In this paper, *t*-test (filter approach) method is used to select the high relevance genes. It assumes independence among genes while determining the rankings and is computationally very efficient.

However, a linear subspace cannot describe the nonlinear variations of microarray genes. Alternatively, a kernel feature space can reflect nonlinear information of genes, in which the original data points are mapped onto a higher-dimensional (possibly infinite-dimensional) feature space defined by a function *ϕ* (usually nonlinear) through a mathematical process called the “kernel trick” [23].

The kernel trick is a mathematical technique which can be applied to any algorithm. It solely depends on the dot product between two vectors. Wherever a dot product is used, it is replaced by the kernel function. When properly applied, these candidate linear algorithms are transformed into nonlinear algorithms (sometimes with little effort or reformulation). These nonlinear algorithms are equivalent to their linear originals operating in the range space of a feature space.

In the literature, it is observed that the following types of kernels have been used to map the function in high dimensional space:

(i) *linear: K*(*x*_*i*_, *x*_*j*_) = *x*_*i*_^*T*^ *x*_*j*_;
(ii) *polynomial: K*(*x*_*i*_, *x*_*j*_) = (*γx*_*i*_^*T*^ *x*_*j*_ + *c*)^*d*^; *γ* > 0, *c*⩾0;
(iii) *radial basis function (RBF): K*(*x*_*i*_, *x*_*j*_) = exp⁡(−*γ*||*x*_*i*_−*x*_*j*_||^2^); *γ* > 0;
(iv) *tan-sigmoid (tansig): K*(*x*_*i*_, *x*_*j*_) = tanh(*γx*_*i*_^*T*^ *x*_*j*_ + *c*); *γ* > 0, *c*⩾0.

where *γ*, *c*, and *d* are* kernel parameters*.

The choice of a kernel function depends on the problem in hand because it depends on what we are trying to model. For instance, a polynomial kernel allows feature conjunction modeling to the order of the polynomial. Radial basis function allows picking out circles (or hyperspheres) in contrast with the linear kernel, which allows only picking out lines (or hyperplanes). The objective behind using the choice of a particular kernel can be very intuitive and straightforward depending on what kind of information is to be extracted with respect to data.

Fuzzy logic provides a means to arrive at a definite conclusion based upon vague, ambiguous, imprecise, noisy, or missing input information. Since the nature of dataset is quite fuzzy, that is, not predictable, which in turn (data) leads to different inference, the relationship among the data and inference is unknown. The fuzzy concept has been used in this work, to study the behavior of the data (capturing human way of thinking), and also it is also possible to represent and describe the data mathematically. Further, fuzzy system has been considered because of the limited number of learning rules that needs to be learnt in the present system. The number of free parameters to be learnt is reduced considerably, leading to efficient computation. In general, if the number of features is larger than 100, then it is suitable to use machine learning techniques rather than using statistical approaches.

If ANN is applied for the same method, designing the model would be far more challenging due to the large number of cases. Hence coupling ANN with Fuzzy logic will be easy to handle by inferring the rule base of the fuzzy system.

In the current scenario, neurofuzzy networks have been found to be successfully applied in various areas of analytics. Two typical types of neurofuzzy networks are Mamdani-type [24] and TSK-type [25]. For Mamdani-type neurofuzzy networks, minimum number of fuzzy implications are used in fuzzy reasoning. Meanwhile, in TSK-type neurofuzzy networks, the consequence of each rule is a function of various input variables. The generic adopted function for rule generation is a linear combination of input variables and constant term. Several researchers and practitioners have reported that using TSK-type neurofuzzy network achieves superior performance in network size and learning accuracy to that of Mamdani-type neuron-fuzzy networks [26]. In classic TSK-type neurofuzzy network, which is linear polynomial of the input variables, the system output is approximated locally by the rule of hyperplanes.

Along with the feature selection using t-statistic, a nonlinear version of FIS called kernel fuzzy inference system (K-FIS) using 10-fold cross-validation (CV). The results obtained from the experimental work carried out on leukemia dataset show that the proposed methods perform well when certain performance indicators are considered.

The rest of the paper is organized as follows.  Section 2 highlights the related work in the field of microarray classification.  Section 3 presents the proposed work for classifying the microarray data using kernel fuzzy inference system (K-FIS).  Section 4 presents the various performance parameters used to evaluate the performance of classifiers (models).  Section 5 gives the details of the implementation work carried out for classification.  Section 6 highlights the results obtained and interpretation drawn from it and also presents a comparative analysis for gene classification of microarray.  Section 7 concludes the paper with scope for future work.

## 2. Related Work

This section gives a brief overview of the feature selection methods and classifiers used by various researchers and practitioners and their respective accuracy rate achieved in gene classification.  Table 1 gives the list of classifiers and features selection/extraction methods.

## 3. Proposed Work

The presence of a huge number of insignificant and irrelevant features degrades the quality of analysis of the disease like “cancer.” To enhance the quality, it is very essential to analyze the dataset in proper perspective. This section presents the proposed approach for classification of microarray data, which consists of two phases:

1. this phase, preprocessess the input data using various methods such as missing data imputation, normalization, and feature selection using* t*-statistic.
2. the fact that K-FIS algorithm has been applied as a classifier.

**Figure 1:**
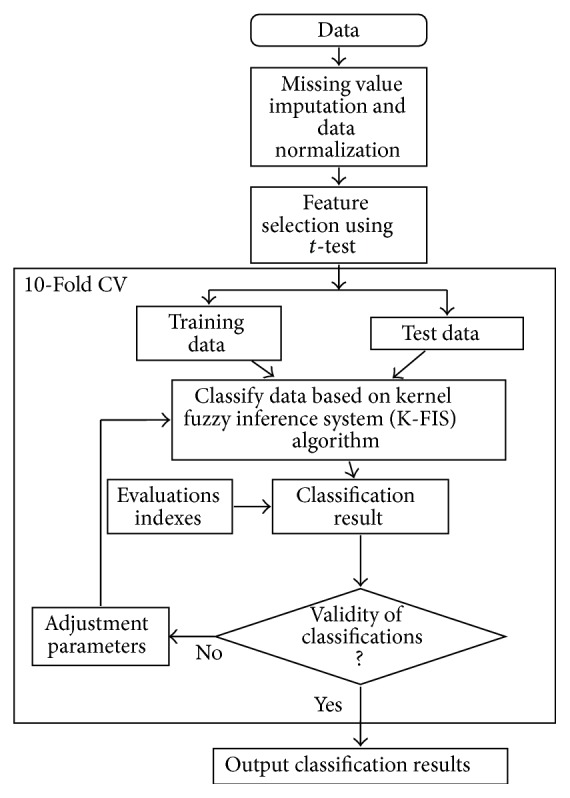
Proposed work for microarray classification.

Figure 1 shows the graphical representation of proposed approach and the brief description of the proposed approach is as follows.

(*1) Data Collection*. The requisite input data for microarray classification is obtained from Kent Ridge Biomedical Dataset Repository [1]. 

(*2) Missing Data Imputation and Normalization of Dataset.* Missing data of a feature (gene) of microarray data is imputed by using the mean value of the respective feature. Input feature values are normalized over the range [0,1] using min-max normalization technique [27]. Let *X*_*i*_ be the *i*_th_ feature of the dataset *X*, and *x* is an element of the *X*_*i*_. The normalization of the *x* can be calculated as

(1) $$\begin{array}{c} \text{Normalized}\left(x\right)=\frac{x-\min\left(X_{i}\right)}{\max\left(X_{i}\right)-\min\left(X_{i}\right)}, \end{array}$$

where min⁡(*X*_*i*_) and max⁡(*X*_*i*_) are the minimum and maximum value for the dataset *X*_*i*_, respectively. If max⁡(*X*_*i*_) is equal to min⁡(*X*_*i*_), then normalized(*x*) is set to 0.5.

(*3) Division of Dataset.* The dataset is divided into two categories such as training set and testing set.

(*4) Feature Selection of Dataset. t*-test statistics has been applied to select the features having high relevance value and hence the curse of dimensionality issue has been reduced.

(*5) Build Classifier.* Kernel fuzzy inference system (K-FIS) has been designed to classify the microarray dataset.

(*6) Test the Model.* Model is tested using the testing dataset and then the performance of the classifier has been compared using various performance measuring criteria based on “10-fold cross-validation” technique.

## 4. Performance Evaluation Parameters

This section describes the performance parameters used for classification [28] (Table 3).  Table 2 shows the classification matrix, from which the values of the performance parameters can be determined.

## 5. Implementation

### 5.1. Feature Selection Using *t*-Test

Generally, the problems with microarray data are (a) “curse of dimensionality,” where numbers of features are much larger than the number of samples, (b) the fact that there are so many features having very less effect on the classification result, and so forth. To alleviate these problems, feature selection approaches are used. In this paper, *t*-test filter approach is used to overcome the problems. Selecting features using *t*-test is to reduce the dimension of the data by finding a small set of important features which can give good classification performance and is computed using (2):

(2) $$\begin{array}{c} \text{TS}\left(i\right)=\frac{{\bar{X}}_{i1}-{\bar{X}}_{i2}}{s_{X_{1}X_{2}}\sqrt{\left(1/n_{1}\right)+\left(1/n_{2}\right)}}, \end{array}$$

(3) $$\begin{array}{c} s_{X_{1}X_{2}}^{2}=\frac{\left(n_{1}-1\right)s_{X_{i1}}^{2}+\left(n_{2}-1\right)s_{X_{i2}}^{2}}{n_{1}+n_{2}-2}, \end{array}$$

where *s*_*X*_1_*X*_2__ is an estimator of the common standard deviation of the two samples, ${\bar{X}}_{ik}$ represents the mean of feature *i* of class *k* ∈ {1,2}, and *s* is the standard deviation.

**Figure 2:**
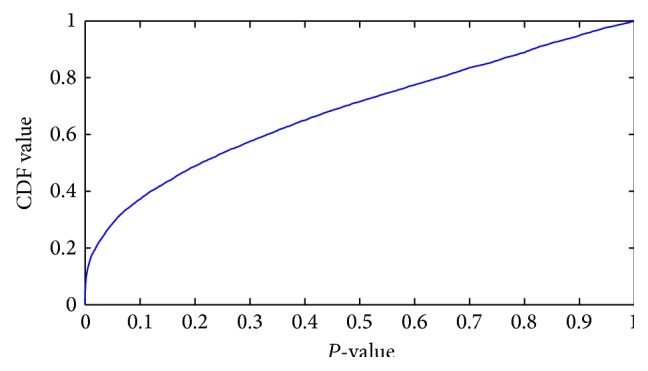
Empirical cumulative distribution function (CDF) of the *P* values.

A widely used filter method for microarray data is to apply a univariate criterion separately on each feature, assuming that there is no interaction between features. A two-class problem test of the null hypothesis (*H*_0_) is that the means of two populations are equal; it means that there is no significant difference between their means, and both features are almost the same. It implies that they (features) do not affect much the classification result. Hence, these features have been discarded, and the features having significant difference between their means are accepted. Therefore, it is necessary to reject “null hypothesis” (*H*_0_) and accept the “alternate hypothesis” (*H*_1_). In other words, alternate hypothesis is accepted. Here, *t*-test on each feature has been applied and compared with their corresponding *P* value (or the absolute values of *t*-statistics) for each feature as a measure of how effective it is at separating groups. In order to get a general idea of how well separated the two groups (classes) are by each feature, the empirical cumulative distribution function (CDF) of the *P* values has been plotted in Figure 2.

From Figure 2, it is observed that about 18% of features are having *P* values close to zero and over 28.70% of features are having *P* values smaller than 0.05. The features having *P* values smaller than 0.05 have strong discrimination power. Sorting these features according to their *P* values (or the absolute values of the *t*-statistic) helps to identify some features from the sorted list. However, it is usually difficult to decide how many features are needed unless one has some domain knowledge or the maximum number of features that can be considered has been dictated in advance based on outside constraints. To overcome this problem, forward feature selection method is considered, in which top ranked features corresponding to their descending *P* value are identified.

### 5.2. Fuzzy Inference System (FIS)

For a given universe set *U* of objects, a conventional binary logic (crisp) *A* is defined by specifying the objects of *U* that are member of *A*. In other words, the characteristic function of *A* can be written as *u*_*A*_ : *U* → {0,1} for all *x* ∈ *U*.

Fuzzy sets are obtained by generalizing the concept of characteristic function to a membership function *u*_*A*_ : *U* → [0,1] for all *x* ∈ *U*. It provides the degree of membership rather than just the binary* is*/*is not* a member to a set, which ensures the objects that are not clearly member of one class or another. Using crisp techniques, an ambiguous object will be assigned to one class only lending an aura of precision and definiteness to the assignments that are not warranted. On the other hand, fuzzy techniques will specify to what degree the object belongs to each class.

The TSK fuzzy model (FIS) is an adaptive rule model introduced by Takagi et al. [25, 26]. The main objective of using TSK fuzzy model is to reduce the number of rules generated by Mamdani model. In this approach, TSK fuzzy model can also be used for classifying complex and high dimensional problems. It develops a systematic approach to generating fuzzy rules from a given input-output dataset. TSK model replaces the fuzzy sets of the Mamdani rule with the function of the input variables.

### 5.3. Kernel Fuzzy Inference System (K-FIS)

**Figure 3:**
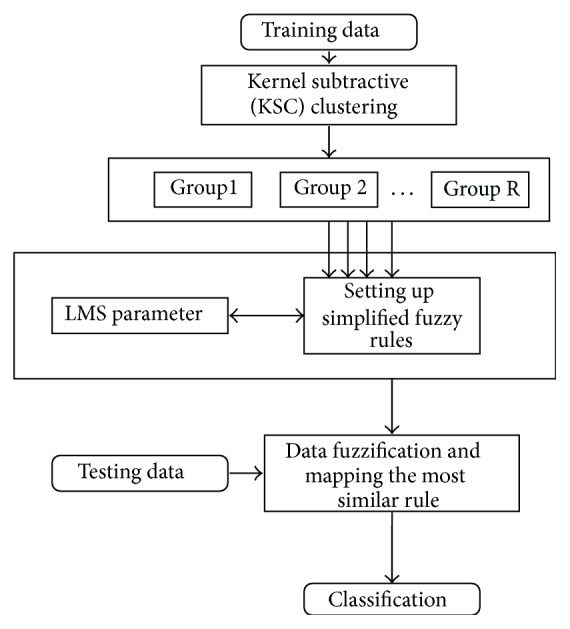
Framework of kernel fuzzy inference system (K-FIS).

In this section, K-FIS has been described which is a nonlinear version of FIS. The number of rules (*R*), the parameters of fuzzy sets, that is, the centers and the width parameters (*σ*) of the corresponding membership function (in this case Gaussian) of K-FIS, are computed using kernel subtractive clustering technique (KSC) which is also a nonlinear version of subtractive clustering (SC) and the parameters of rules are computed using least mean square (LMS) in nonlinear space. The stepwise working procedure of K-FIS has been depicted in Figure 3. The working procedure of K-FIS is described as follows.

(*1) Clustering.* To compute the parameters of the membership function, that is, centroids and sigmas (*σ*) and number of rules (centers), Kernel subtractive clustering (KSC) has been used on training dataset (microarray). The algorithm of KSC has been described in Section 5.3.1.

(*2) Setting Up a Simplified Fuzzy Rule Base.*

*(i) Computation of Membership Function.* Gaussian function is used as a membership function (*A*). The parameters such as centroid (*c*) and sigma (*σ*) of *A* have been computed using KSC and *A* is expressed as

(4) $$\begin{array}{c} A=\exp\left(-\frac{1}{2}{\left(\frac{x-c}{\sigma }\right)}^{2}\right). \end{array}$$

*(ii) Generation of Fuzzy Rules.* The number of fuzzy rules generated will be equal to the number of clusters formed.

(*3) Estimation of Parameters of Rules.* After generating fuzzy rules, the constant parameters in rules can be estimated using least mean square (LMS) algorithm.

#### 5.3.1. Kernel Subtractive Clustering (KSC)

The kernel subtractive clustering (KSC) is a nonlinear version of subtractive clustering [29]; here input space is mapped into nonlinear space. In this algorithm, to obtain the cluster centroids and sigmas, the same parameters are used which are also used in subtractive clustering (SC) [30]. The parameters used to calculate the cluster centroid are* Hypersphere cluster radius (r*_*a*_ *) in data space*,* reject ratio (* $\bar{\epsilon }$),* accept ratio (ϵ*). Squash factor (*η*) defines the neighborhood which will have the measurable reductions in potential value, and it can be calculated as

(5) $$\begin{array}{c} \eta =\frac{r_{b}}{r_{a}}. \end{array}$$

Reject ratio ($\bar{\epsilon }$) specifies a threshold for the potential value above which the data point is definitely accepted as a cluster centroid. Accept ratio (*ϵ*) specifies a threshold below which the data point is definitely rejected.

For a given data point *x*_*i*_ ⊂ *X* where (1 ⩽ *i* ⩽ *n*), *X* ∈ *ℝ* ^*p*^, and a nonlinear function *ϕ*, *ℝ* ^*p*^ → *ℍ* maps the input to a higher- (may be infinite-) dimensional feature space *ℍ*. The potential value of each data point defines a measure of the data point to serve as a cluster centroid and can be calculated by using the following equation:

(6) p(xi)=∑j=1ne−α||ϕ(xi)−ϕ(xj)||2=∑j=1ne−α(K(xi,xi)−2K(xi,xj)+K(xj,xj)),

where *α* = 4/*r*_*a*_^2^, *K* is a kernel function, ||·|| denotes the Euclidean distance between the data points, and *r*_*a*_ is a positive constant called cluster radius. The data point with highest potential is selected as the first cluster centroid by computing the potential value of individual data point. Let *x*_1_^*^ be the centroid of the first cluster and *p*_1_^*^ its potential value. The potential value of each data point *x*_i_^*^ is revised as follows:

(7) pj(xi)=pj−1(xi)−pj−1∗e−β||ϕ(xi)−ϕ(xj−1∗)||2=pj−1(xi)−pj−1∗e−β(K(xi,xi)−2K(xi,xj−1∗)+K(xj−1∗,xj−1∗)),

where *p*_j_^*^ = *Max*⁡_*i*_(*p*(*x*_*i*_)), *β* = 4/*r*_*b*_^2^, *r*_*b*_ = *η*∗*r*_*a*_, and *η* is a positive constant over the range [1,2]. When the potentials of all data points have been revised by (7), the data point with the highest remaining potential is selected as the second cluster centroid. In such a manner, all the cluster centroids are selected using Algorithm 1.

After computing the number of rules (*R*), the parameters of fuzzy sets and the parameters of rules are derived. To derive the rules for the K-FIS, the selected features (genes) using filter approach (*t*-test) have been used as the input. The *k* ^th^ rule (*R* ^*k*^) for the given test point *x*_*t*_ can be expressed as.

IF *x*_1_ is *A*_1_^*k*^ and *x*_2_ is *A*_2_^*k*^,… and *x*_*n*_ is *A*_*n*_^*k*^,

where *x*_1_, *x*_2_,…, *x*_*n*_ are input variables and *A*_*j*_^*k*^ is a fuzzy set, *R* ^*k*^ is a linear function. The fuzzy set *A*_*j*_^*k*^ uses a Gaussian function and can be computed as

(8) Ajk=exp⁡(−12(ϕ(xj)−ϕ(cjk)σjk)2)=exp⁡(−12σjk2(K(xj,xj)−2K(xi,cjk)+K(cjk,cjk)))

THEN

(9) $$\begin{array}{c} R^{k}=b_{0}^{k}+\overset{n}{\underset{t=1}{\sum }}p_{t}^{k}\phi \left(x_{t}\right) \end{array}$$

(10) $$\begin{array}{c} p_{t}^{k}=\overset{m}{\underset{i=1}{\sum }}\alpha_{i}\phi \left(x_{i}\right). \end{array}$$

Consider *m* to be the number of training samples and *ϕ* as a nonlinear transformation function. The representer theorem [31, 32] states that the solution of an optimization of (10) can be written in the form of an expansion over training pattern, (*x*_*i*_ is replaced by *ϕ*(*x*_*i*_)). Therefore, each training vector lies in the span of *ϕ*(*x*_1_), *ϕ*(*x*_2_),…, *ϕ*(*x*_*m*_), and Lagrange multiplier *α*_*i*_, where *i* = 1,2,…, *m* [33]. Therefore, (9) is expressed as

(11) Rk(xt)=b0k+∑t=1n∑i=1mαiϕ(xi)ϕ(xt)=b0k+∑t=1n∑i=1mαiK(xi,xt).

The degree (firing strength) with which the input matches *k* ^th^ rule is typically computed using “*and*” operator:

(12) $$\begin{array}{c} \mu_{k}=\overset{n}{\underset{j=1}{\prod }}A_{j}^{k}. \end{array}$$

In this case, each rule is a crisp output. The overall output is calculated using the weighted average as shown in the following:

(13) $$\begin{array}{c} Y=\frac{\sum_{i}^{R}\mu_{i}R_{i}}{\sum_{i}^{R}\mu_{i}}, \end{array}$$

where *R* is the number of rules and *R*_*i*_ is the *i* ^th^ fuzzy rule where *i* = 1,2,…, *R*. For K-FIS classification algorithm, the probability $\hat{y}$ of output *Y* can be calculated using the following [34]:

(14) $$\begin{array}{c} \hat{y}={\left(1+\exp\left(-Y\right)\right)}^{-1}. \end{array}$$

Using the usual kernel trick, the inner product can be substituted by kernel functions satisfying Mercer's condition. Substituting the expansion of *p* in (10) into (9), this transformation leads to nonlinear generalization of fuzzy inference system in kernel space which can be called as kernel fuzzy inference system (K-FIS).

## 6. Results and Interpretation

In this section, the obtained results are discussed for the proposed algorithm (Section 3) on a case study, namely, leukemia microarray dataset [1]. The classification performance is assessed using the “10-fold cross-validation (CV)” technique for leukemia dataset. 10-fold CV provides more realistic assessment of classifiers, which generalizes significantly to unseen data.

### 6.1. Case Study: Leukemia

The leukemia dataset consists of expression profiles of 7129 features (genes), categorized as acute lymphoblastic leukemia (ALL), and acute myeloid leukemia (AML) classes, having 72 samples [1]. Out of seventy-two samples, the dataset contains twenty-five (25) AML and forty-seven (47) ALL samples.  Table 4 shows the classification matrix before the application of the classification algorithm.

Since the dataset contains a very large number of features with irrelevant information, feature selection (FS) method has been applied to select the features (genes) which have high relevance score, and the genes with a low relevance score are discarded. *t*-test method has been used to choose genes with high relevance score. The main objectives of the FS method are as follows:

- to avoid overfitting and improve model (classifier) performance,
- to provide faster and more cost-effective models,
- to gain a deeper insight into the underlying processes that generate the data.

To achieve these objectives of FS, forward selection method has been employed by selecting the features having high “*P* value” using *t*-test. The forward selection method has been slightly modified where features are selected in multiples of five; that is, five features are selected corresponding to top five “*P* values” and so on. The selected features are tabulated in Table 5.

After feature selection using *t*-test, the proposed classification algorithm K-FIS is applied to classify the reduced leukemia dataset using 10-fold CV.

The dataset is divided into different subsets for the training and testing purpose. First of all, every tenth sample out of seventy-two (72) samples is extracted for testing purpose and the rest of the data will be used for training purpose. Then the training set has been partitioned into the learning and validation sets in same manner as shown below.

 
*For partition 1.* Samples 1,11,21,… are used as validation samples and the remaining are accepted as learning samples.
 
*For partition 2.* Samples 2, 12, 22, … are used as validation samples and the remaining are accepted as learning samples.
 ⋮
 
*For partition 10.* Samples 10, 20, 30, … are used as validation samples and the remaining are accepted as learning samples.

After partitioning data into learning set and validation set, model selection is performed using 10-fold CV process by varying the parameters of K-FIS. The parameters used in the proposed work are shown in Table 6.

By varying the value of *r*_*a*_, the best model (with high accuracy or minimum error) is selected in each fold using Algorithm 2, where *F* represents the number of folds which is equal to ten.

### 6.2. Interpretation of Results

After feature selection using *t*-test, K-FIS has been used as a classifier to classify the microarray dataset by performing 10-fold CV. Different number of features set, namely, 5, 10, 15, and so on, have been considered and then their corresponding training (training data) and testing accuracies (using testing data) are computed.

#### 6.2.1. Analysis of Kernel Fuzzy Inference System (K-FIS)

In this study, kernel TSK fuzzy (K-FIS) approach based on kernel subtractive clustering (KSC) has been used to classify the microarray gene expression data. The process of classifier (model) building using KSC has been carried out by formation of clusters in the data space and translation of these clusters into TSK rules. The number of clusters signifies the number of rules; that is, the number of rules in K-FIS will be equal to a number of clusters obtained using KSC. The parameters used in K-FIS are shown in Table 6 and the value of *r*_*a*_ has been optimized using cross-validation and results are computed.

After feature selection using *t*-test, the features are taken in a set of 5, 10, 15, 20, 25, and 30 called F5, F10, F15, F20, F25, and F30 (shown in Table 5), respectively, as an input to the classifier K-FIS and corresponding to that input vector performance of classifier has been analyzed. The K-FIS has been implemented using various kernel functions, namely, linear, polynomial, RBF, and tansig.

(*1) Analysis of K-FIS Using Linear Kernel (L-FIS).* As a nonlinear version of FIS, K-FIS is more general model and contains FIS as an instance when the linear kernel is employed. Figures 9 and 10 show the comparison of accuracy obtained in each fold using training data and testing data by considering varying number of features like 5, 10, 15, 20, 25, and 30, respectively, shown in the appendix.

**Figure 4:**
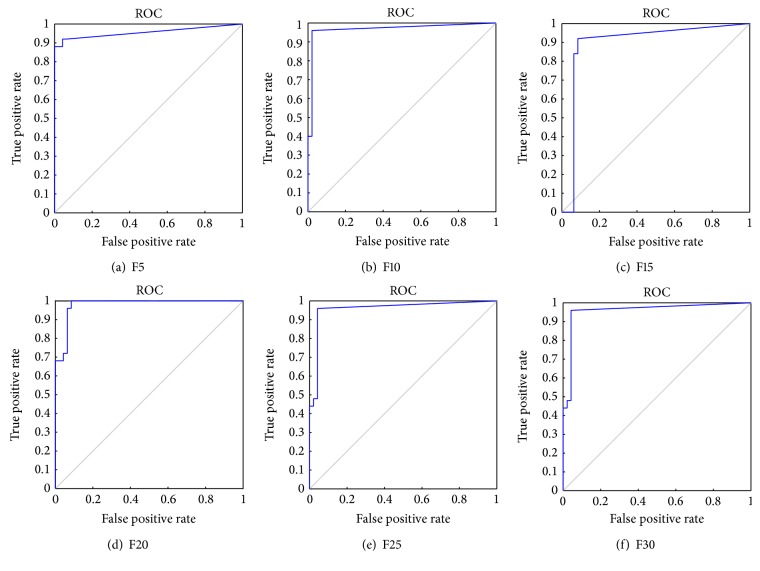
ROC curve for FIS with different set of features.

After performing “10-fold CV” on the dataset, the predicted values of test data are collected from each of the folds and classification matrix has been computed in each of the cases as shown in Table 7. For instance, in model F5, five (5) features are selected, and then classification is performed. Tables 4 and 7(a) represent the classification matrix for number of classes with ALL and AML, before and after applying L-FIS classifier, respectively. It is evident that, before applying the L-FIS, out of 72 samples; 47 samples were classified as ALL class and the rest 25 samples are classified into AML class. But after applying L-FIS (with F5) analysis, it is found that a total number of 67 (23 (AML) + 44(ALL)) samples are classified correctly with an accuracy rate of 93.06%. Similarly, using L-FIS with a different set of features, namely, F10, F15,…, F30, the classification matrix has been tabulated in Tables 7(b), 7(c), 7(d), 7(e), and 7(f), respectively, and their ROC curve plots are shown in Figure 4.  Table 8 shows the value of cluster radius *r*_*a*_ (i.e., the median of the best value of *r*_*a*_ in each fold) and the value of various performance parameters used to evaluate the performance of model for classification.

It has been observed that L-FIS as a classifier achieved highest accuracy when 10 numbers of features (i.e., F10) have been selected. Model L-FIS has high (*Recall * = 96%) capacity to identify relevant item and also to identify negative labels (*Specificity* = 97.87%) in case of F10.

Hence from the obtained results, it is concluded that the role of feature selection is very important to classify the data with the classifier.

(*2) Analysis of K-FIS Using Polynomial Kernel (P-FIS).* Figures 11 and 12 show the comparison of accuracy obtained in each fold using training data and testing data by taking different number of features, namely, 5, 10, 15, 20, 25, and 30, respectively, has been shown in the appendix. After performing “10-fold CV” on the dataset, the predicted values of test data are collected from each of the folds and classification matrix has been computed in each of the cases as shown in Table 9 and different performance measuring parameters are computed. For instance, K-FIS with F5 model, five (5) features are selected, and then classification is performed.

The *gamma*(*γ*) value of polynomial kernel is selected by searching in the range of each fold, that is, 2^−5^ to 2^5^. Finally, the median value of the best *gamma* from each fold is considered as the value of *gamma* for the final model.

**Figure 5:**
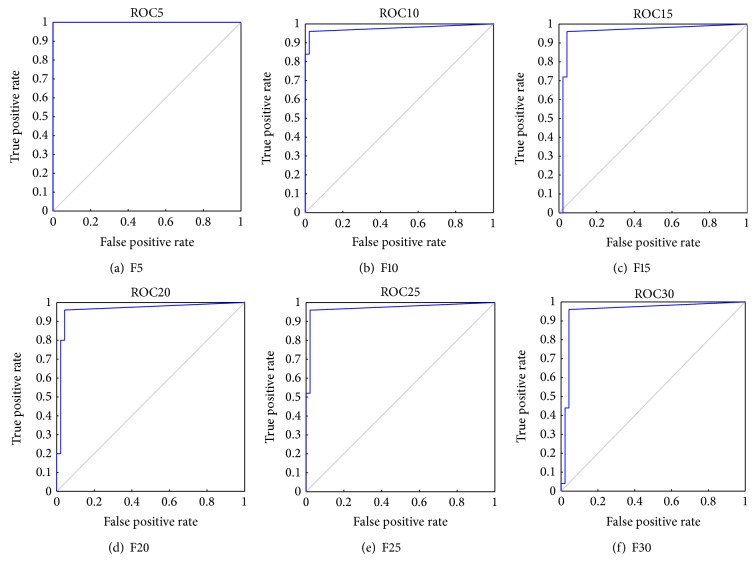
ROC curve for K-FIS using polynomial kernel (*γ* = 1, *c* = 0.5, and *d* = 3) with various feature sets.

In comparison with Table 4 K-FIS was able to classify a total of 71 (25 (AML) + 46 (ALL)) classes with respect to F5 by obtaining 98.61% of accuracy. Similarly, using K-FIS with a different set of features, namely, F10, F15,…, F30, the classification matrix has been tabulated in Tables 9(b), 9(c), 9(d), 9(e), and 9(f), respectively, and the obtained ROC curves have been shown in Figure 5.

After analyzing K-FIS (polynomial) with various sets of features, Table 10 shows the value of cluster radius *r*_*a*_ (i.e., the median of the best value of *r*_*a*_ in each fold) and the value of various performance parameters used to evaluate the performance of model for classification. It is observed that, K-FIS (P-FIS) classifier achieved the highest accuracy with 98.61% when 5 numbers of features (i.e., F5) have been selected. Model polynomialhas high (*Recall * = 96.15%) capacity to identify relevant items and also to identify negative labels (*Specificity * = 100%) in case of F5, when compared with other feature sets of K-FIS. Hence, from the obtained results, it can be concluded that the role of feature selection is very significant in order to classify the microarray dataset.

(*3) Analysis of K-FIS Using RBF Kernel (R-FIS).* Figures 13 and 14 show the comparison of accuracy obtained in each fold using training data and testing data by taking different number of features, namely, 5, 10, 15, 20, 25, and 30, respectively; vide the appendix.

After performing “10-fold CV” on the dataset, the predicted values of test data are collected from each of the folds and classification matrix has been computed in each of the cases as shown in Table 11 and different performance measuring parameters are computed. For instance, K-FIS with F5, five (5) features are selected, and then classification is performed. The *gamma*(*γ*) value of RBF kernel is selected by searching in the range of each fold, that is, 2^−5^ to 2^5^. Finally, the median value of the best *gamma* from each fold is considered as the value of *gamma* for the final model.

**Figure 6:**
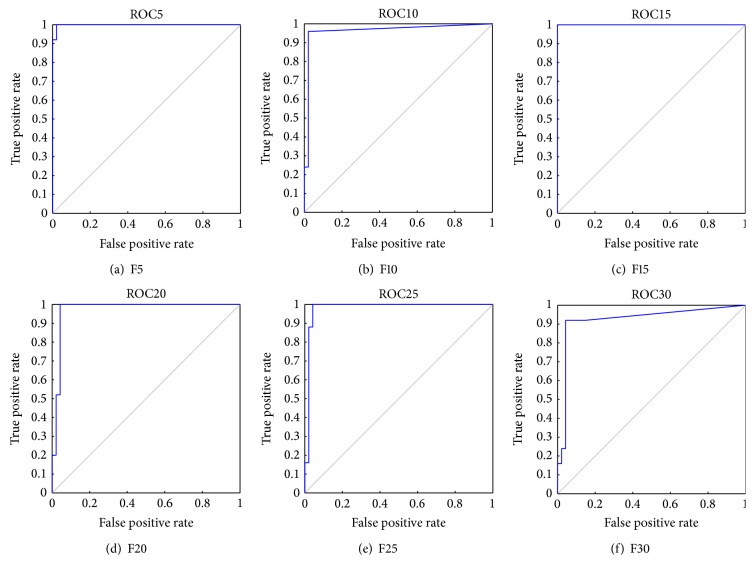
ROC curve for K-FIS using RBF kernel (*γ* = 0.5) with various feature sets.

In comparison with Table 4 K-FIS was able to classify a total of 70 (25 (AML) + 45 (ALL)) classes with respect to F5 by obtaining 97.22% of accuracy. Similarly, using K-FIS with a different set of features, namely, F10, F15,…, F30, the classification matrix has been tabulated in Tables 11(b), 11(c), 11(d), 11(e), and 11(f), respectively, and the obtained ROC curves have been shown in Figure 6.

After analyzing K-FIS (RBF) with various sets of features, Table 12 shows the value of cluster radius *r*_*a*_ (i.e., the median of the best value of *r*_*a*_ in each fold) and the value of various performance parameters used to evaluate the performance of model for classification. It is observed that K-FIS (RBF) classifier achieved highest accuracy with 97.22% when 5 numbers of features (i.e., F5) are selected. Model R-FIS has high (*Recall * = 92.59%) capacity to identify relevant items and also to identify negative labels (*Specificity* = 100%) in case of F5, when compared with other feature sets of R-FIS. Hence, from the obtained results, it is concluded that the role of feature selection is very important to classify the data with the classifier.

(*4) Analysis of K-FIS Using Tansig Kernel (T-FIS).* Figures 15 and 16 show the comparison of accuracy obtained in each fold using training data and testing data by taking different number of features, namely 5, 10, 15, 20, 25, and 30, respectively, as shown in the appendix.

After performing “10-fold CV” on the dataset, the predicted values of test data are collected from each of the folds and classification matrix has been computed in each of the cases as shown in Table 11 and different performance measuring parameters are computed. For instance, K-FIS with F5 in the model, F5 five (5) features are selected, and then classification is performed. The *gamma*(*γ*) value of tansig kernel is selected by searching in the range of each fold, that is, 2^−5^ to 2^5^. Finally, the median value of the best *gamma* from each fold is considered as the value of *gamma* for the final model.

**Figure 7:**
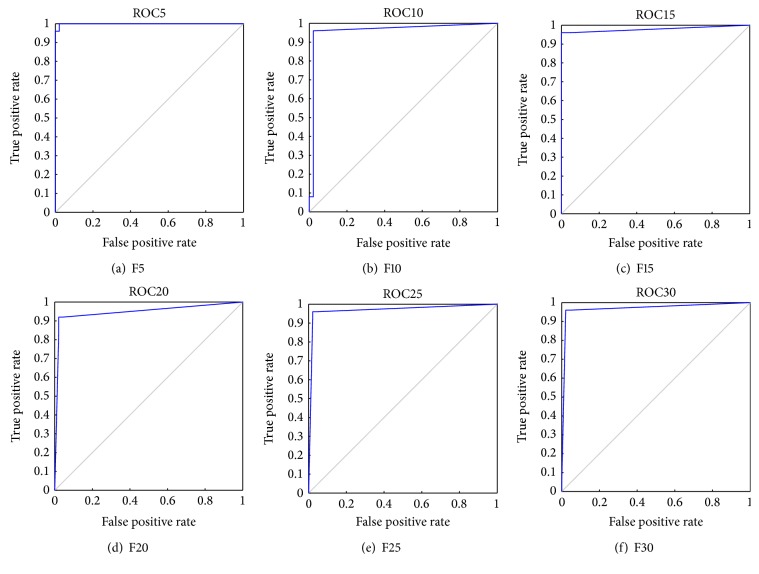
ROC curve for K-FIS using tansig kernel (*γ* = 0.5, *c* = 0.1) with various feature sets.

In comparison with Table 4 K-FIS was able to classify a total of 71 (25 (AML) + 46 (ALL)) classes with respect to F5 by obtaining 98.61% of accuracy. Similarly, using K-FIS with a different set of features, namely, F10, F15,…, F30, the classification matrix has been tabulated in Tables 13(b), 13(c), 13(d), 13(e), and 13(f), respectively, and the obtained ROC curves have been shown in Figure 7.

After analyzing K-FIS (Tansig) with various sets of features, Table 14 shows the value of cluster radius *r*_*a*_ (i.e., the median of the best value of *r*_*a*_ in each fold) and the value of various performance parameters used to evaluate the performance of model for classification. It has been observed that K-FIS (Tansig) classifier achieved highest accuracy with 98.61% when 5 numbers of features (i.e., F5) had been selected. Model T-FIS has high (*Recall * = 96.15%) capacity to identify relevant item and also to identify negative labels (*Specificity* = 100%) in case of F5 comparison to K-FIS with other sets of features. In case of F10, accuracy is 97.22% and accuracies of K-FIS with F15, F25, and F30 are the same with 95.83%. Since the variation of classifier performance is very flexible, it is concluded that the role of feature selection is very important to classify the data with the classifier.

### 6.3. Comparative Analysis

A best model for classification of microarray data is chosen based on the performance parameters such as accuracy, precision, recall, specificity, and *F*-measure. The values obtained for the respective parameters are shown in Table 15. The results of proposed algorithm are compared with the SVM classifier. From Table 15, the following can be inferred that.

(i) In case of K-FIS classification using different kernel functions, tansig kernel function obtained high values of accuracy with different set of features, namely, F5, F10, F15, F20, F25, and F30. The respective accuracies for the features are 98.61%, 97.22%, 95.83%, 94.44%, 96.83% and 96.83% respectively on test data.
(ii) In case of SVM classifier with different kernel functions:
  (1) the parameters of the kernel functions like *gamma*(*γ*) and the penalty parameter *C* are selected using the grid search in the range of [2^−5^, 2^5^] and [2^−5^, 2^5^], respectively,
  (2) from Table 15, it is observed that 100% testing accuracy is achieved (for F15), when SVM is used along with RBF kernel.

**Figure 8:**
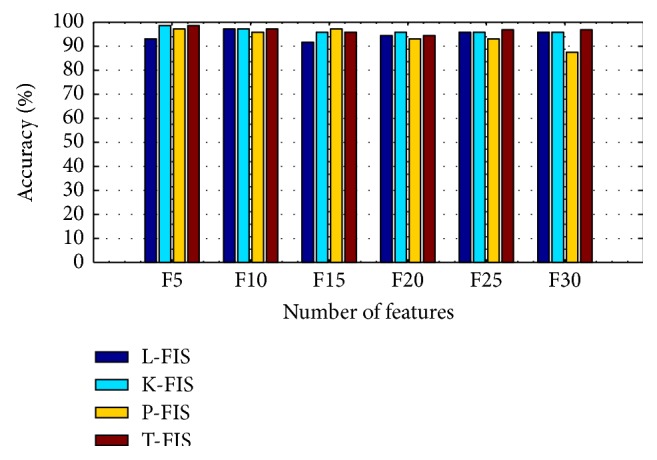
Comparison of testing accuracy of K-FIS using different feature set.

**Figure 9:**
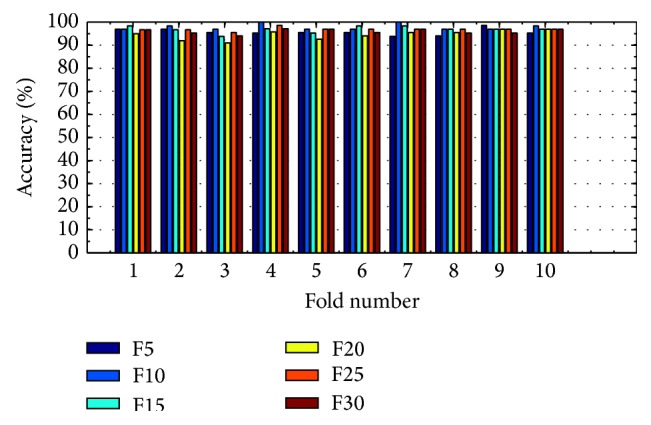
Training accuracy in each fold with different set of features using K-FIS with linear kernel.

**Figure 10:**
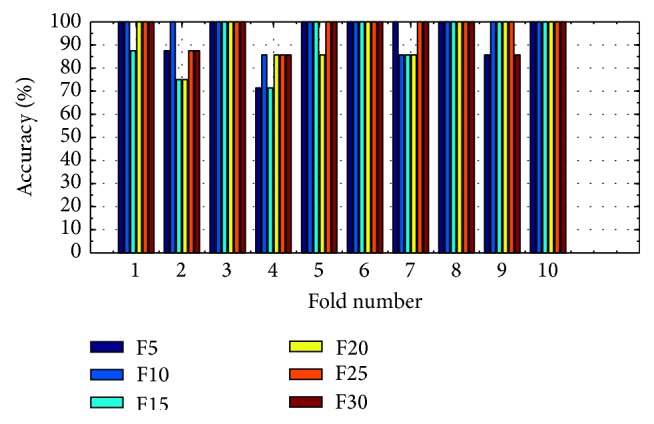
Testing accuracy in each fold with different set of features using K-FIS with linear kernel.

**Figure 11:**
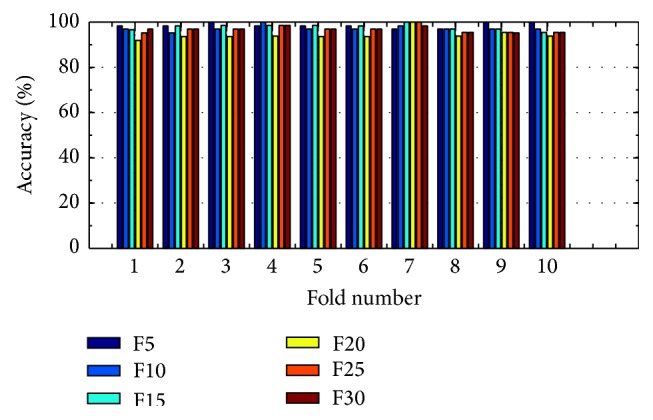
Training accuracy in each fold with different set of features using K-FIS with polynomial kernel.

**Figure 12:**
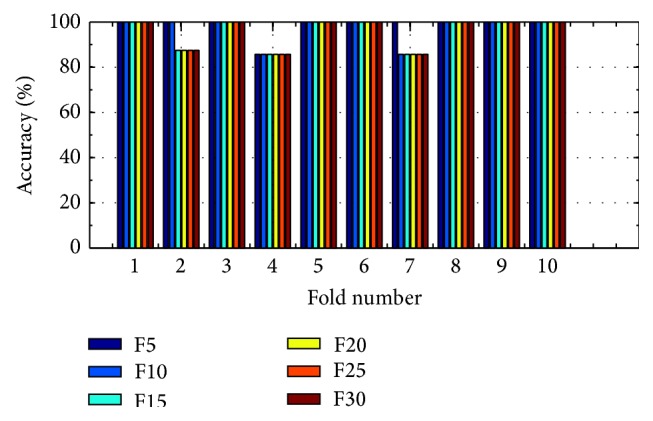
Testing accuracy in each fold with different set of features using K-FIS with polynomial kernel.

**Figure 13:**
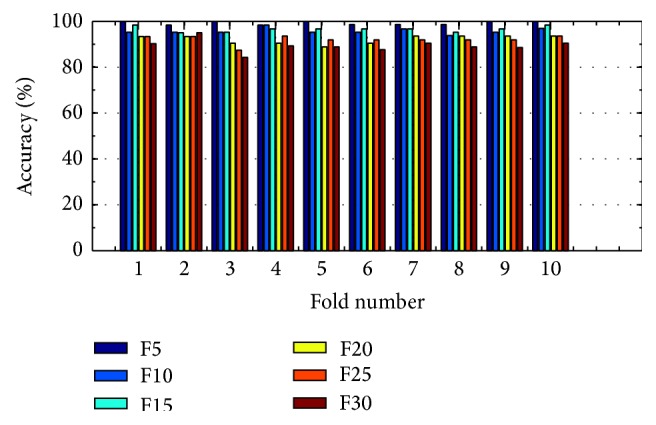
Training accuracy in each fold with different set of features using K-FIS with RBF kernel.

**Figure 14:**
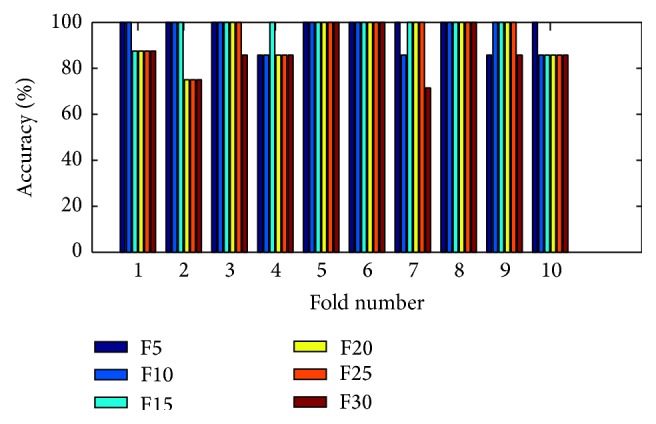
Testing accuracy in each fold with different set of features using K-FIS with RBF kernel.

**Figure 15:**
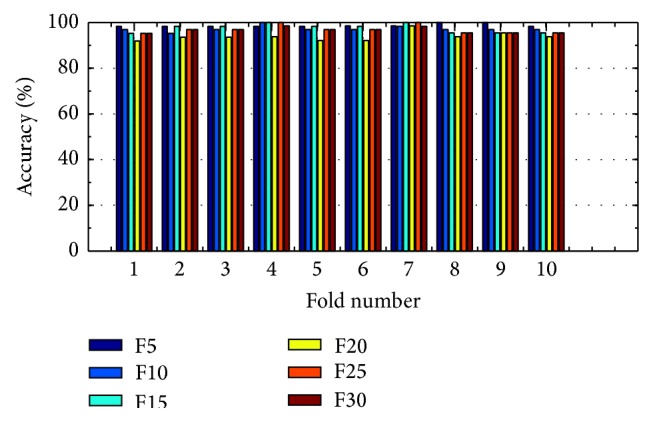
Training accuracy in each fold with different set of features using K-FIS with tansig kernel.

**Figure 16:**
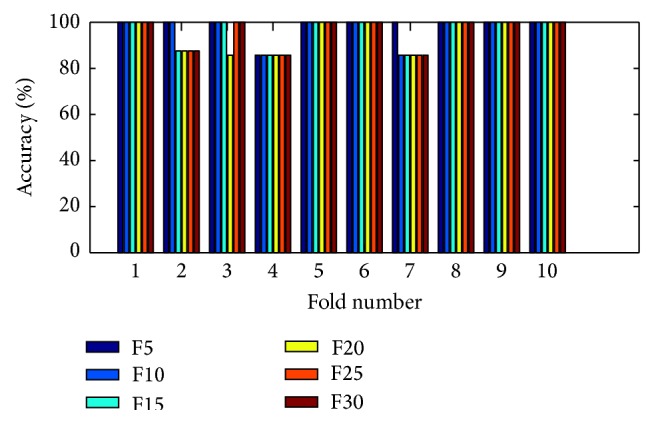
Testing accuracy in each fold with different set of features using K-FIS with tansig kernel.

The comparative analysis of the accuracies of different models has been presented in Figure 8. Based on the performance parameter, it can be concluded that, out of two classifiers, that is, K-FIS and SVM for microarray data classification, K-FIS with tansig kernel method and SVM with RBF kernel yielded better performance.

The running time of the classification algorithm depends on number of features (genes) and number of training data points. The running times were recorded using MATLAB'13a on Intel Core(TM) i7 CPU with 3.40 GHz processor and 4 GB RAM for different models in Table 15 (within small braces).

## 7. Conclusion

In this paper, an attempt has been made to design a classification model for classifying the samples of leukemia dataset either into ALL or AML class. In this approach, a framework was designed for construction of K-FIS model. K-FIS model was developed on the basis of KSC technique in order to classify the microarray data using “kernel trick.” The performance of the classifier for leukemia dataset was evaluated by using 10-fold cross-validation.

From the computed result, it is observed that K-FIS classifier using different kernels yields very competitive result than SVM classifier. Also, when the overall performance is taken into consideration, it is observed that tansig kernel coupled with K-FIS classifier acts as a more effective classifier among the selected classifiers in this analysis. It is evident from the obtained results that “kernel trick” provides a simple but powerful method for classification where data is nonlinearly separable. Data existing in nonlinear space can be easily classified by using a kernel trick.

Further, kernel trick can be applied for all the existing classifiers or to the recently proposed classifiers to classify the data with high predictive accuracy.

## Figures and Tables

**Algorithm 1:**
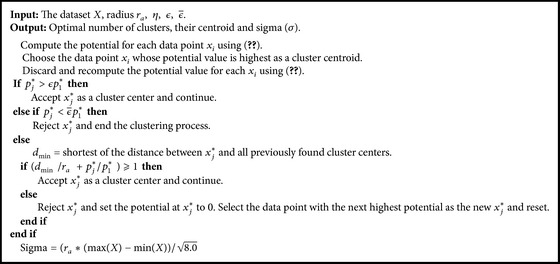
Kernel subtractive clustering.

**Algorithm 2:**
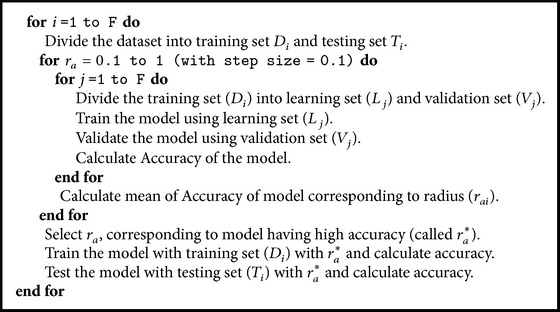
*F*-fold cross-validation.

**Table 1:** Relevant works on cancer classification using microarray (leukemia) dataset.

Author	Feature selection/extraction method	Classifier used	Accuracy (%)
Cho et al. [8] (2003)		Kernel fisher feature discriminant analysis (KFDA)	73.53

Deb and Raji Reddy [9] (2003)		NSGA-II	100

Lee et al. [10] (2003)	Bayesian model	Artificial neural network (ANN), KNN, and SVM	97.05

Ye et al. [11] (2004)	Uncorrelated linear discriminant analysis (ULDA)	KNN (*k* = 1)	97.5

Cho et al. [12] (2004)	SVM-RFE	Kernel KFDA	94.12

Paul and Iba [13] (2004)	Probabilistic model building genetic algorithm (PMBGA)	Naive-Bayes (NB), weighted voting classifier	90

D$\overset{´}{\text{ı}}$az and De andres [14] (2006)		Random forest	95

Peng et al. [15] (2007)	Fisher ratio	NB, decision tree J4.8, and SVM	100, 95.83, and 98.6

Pang et al. [16] (2007)	Bootstrapping consistency gene selection	KNN	94.1

Hernandez et al. [17] (2007)	Genetic algorithm (GA)	SVM	91.5

Zhang and Deng [18] (2007)	Based Bayes error filter (BBF)	Support vector machine (SVM), *K*-nearest neighbor (KNN)	100, 98.61

Bharathi and Natarajan [19] (2010)	ANOVA	SVM	97.91

Tang et al. [20] (2010)	ANOVA	Discriminant Kernel partial least square (Kernel-PLS)	100

Mundra and Rajapakse [7] (2010)	*t*-test, SVM based *t*-statistics, SVM with recursive feature elimination (RFE), and SVM based *t*-statistic with RFE	SVM	96.88, 98.12, 97.88, and 98.41

Lee and Leu [21] (2011)	*χ* ^2^-test	Hybrid with GA + KNN and SVM	100

Salem et al. [22] (2011)	Multiple scoring gene selection technique (MGS-CM)	SVM, KNN, and linear discriminant analysis (LDA)	90.97

**Table 2:** Classification matrix.

	NO	YES
NO	True Negative (TN)	False Positive (FP)
YES	False Negative (FN)	True Positive (TP)

**Table 3:** Performance parameters.

Performance parameters	Description
Precision = TP/(FP + TP)	It is the degree to which the repeated measurements under unchanged conditions show the same results
Recall = TP/(FN + TP)	It indicates that the number of the relevant items are to be identified
*F*-measure = (2∗Precision∗Recall)/(Precision + Recall)	It combines the “precision” and “recall” numeric values to give a single score, which is defined as the harmonic mean of the precision and recall
Specificity = TN/(FP + TN)	It focuses on how effectively a classifier identifies negative labels
Accuracy = (TP + TN)/(FP + FN + TP + TN)	It measures the percentage of inputs in the test set that the classifier correctly labeled
Receive operating characteristic (ROC) curve	ROC curve is a graphical plot which illustrates that the performance of a binary classifier system as its discrimination threshold is varied. It investigates and employs the relationship between “true positive rate (sensitivity)” and “false positive rate (1 − specificity)” of a classifier

**Table 4:** Classification matrix before classification.

	ALL(0)	AML(1)
ALL(0)	47	0
AML(1)	25	0

**Table 5:** Selected features with “*P* value” in descending order.

Number of features	Notation	Selected features with gene ID.
5	F5	{*f*4328, *f*2354, *f*6855, *f*6281, *f*2642}
10	F10	F5 ∪{*f*6225, *f*1144, *f*1685, *f*2335, *f*2441}
15	F15	F10 ∪{*f*6974, *f*804, *f*5772, *f*4973, *f*7119}
20	F20	F15 ∪{*f*6702, *f*758, *f*1962, *f*1928, *f*4196}
25	F25	F20 ∪{*f*5501, *f*4847, *f*4438, *f*5377, *f*4167}
30	F30	F25 ∪{*f*1078, *f*5593, *f*3252, *f*1630, *f*6283}

**Table 6:** Parameters of K-FIS model.

Parameters used	Range	Value used
Squash factor (*η*)	[1, 2]	1.25
Accept ratio (*ϵ*)	(0, 1]	0.75
Reject ratio ($\overset{-}{\epsilon }$)	(0, 1]	0.15
Cluster radius (*r*_*a*_)	(0, 1]	—

**Table:** (a) F5

	0	1
0	44	3
1	2	23

**Table:** (b) F10

	0	1
0	46	1
1	1	24

**Table:** (c) F15

	0	1
0	43	4
1	2	23

**Table:** (d) F20

	0	1
0	44	4
1	0	25

**Table:** (e) F25

	0	1
0	45	2
1	1	24

**Table:** (f) F30

	1	0
0	45	2
1	1	24

**Table 8:** Performance analysis of FIS with different set of features with best suitable cluster radius (*r*_*a*_ in small bracket).

Models (*r*_*a*_)	Accuracy	Precision	Recall	Specificity	*F*-measure
F5 (0.5)	0.9306	0.9200	0.8846	0.9565	0.9020
F10 (0.2)	0.9722	0.9600	0.9600	0.9787	0.9600
F15 (0.4)	0.9167	0.9200	0.8519	0.9556	0.8846
F20 (0.45)	0.9444	1.0000	0.8621	1.0000	0.9259
F25 (0.2)	0.9583	0.9600	0.9231	0.9783	0.9412
F30 (0.4)	0.9583	0.9600	0.9231	0.9783	0.9412

**Table:** (a) F5

	0	1
0	46	1
1	0	25

**Table:** (b) F10

	0	1
0	46	1
1	1	24

**Table:** (c) F15

	0	1
0	45	2
1	1	24

**Table:** (d) F20

	0	1
0	45	2
1	1	24

**Table:** (e) F25

	0	1
0	45	2
1	1	24

**Table:** (f) F30

	0	1
0	45	2
1	1	24

**Table 10:** Performance analysis of K-FIS using polynomial kernel (*γ* = 1, *c* = 0.5, *d* = 3) with different set of features with best suitable cluster radius (*r*_*a*_ in small bracket).

Models (*r*_*a*_)	Accuracy	Precision	Recall	Specificity	*F*-measure
F5 (0.2)	0.9861	1.0000	0.9615	1.0000	0.9804
F10 (0.2)	0.9722	0.9600	0.9600	0.9787	0.9600
F15 (0.3)	0.9583	0.9600	0.9231	0.9783	0.9412
F20 (0.2)	0.9583	0.9600	0.9231	0.9783	0.9412
F25 (0.2)	0.9583	0.9600	0.9231	0.9783	0.9412
F30 (0.4)	0.9583	0.9600	0.9231	0.9783	0.9412

**Table:** (a) F5

	0	1
0	45	2
1	0	25

**Table:** (b) F10

	0	1
0	45	2
1	1	24

**Table:** (c) F15

	0	1
0	45	2
1	0	25

**Table:** (d) F20

	0	1
0	42	5
1	0	25

**Table:** (e) F25

	0	1
0	42	5
1	0	25

**Table:** (f) F30

	0	1
0	40	7
1	2	23

**Table 12:** Performance analysis of K-FIS using RBF kernel (*γ* = 0.5) with different set of features with best suitable cluster radius (*r*_*a*_ in small bracket).

Models	Accuracy	Precision	Recall	Specificity	*F*-measure
F5 (0.4)	0.9722	1.0000	0.9259	1.0000	0.9615
F10 (0.2)	0.9583	0.9600	0.9231	0.9783	0.9412
F15 (0.3)	0.9722	1.0000	0.9259	1.0000	0.9615
F20 (0.4)	0.9306	1.0000	0.8333	1.0000	0.9091
F25 (0.6)	0.9306	1.0000	0.8333	1.0000	0.9091
F30 (0.6)	0.8750	0.9200	0.7667	0.9524	0.8364

**Table:** (a) F5

	0	1
0	46	1
1	0	25

**Table:** (b) F10

	0	1
0	46	1
1	1	24

**Table:** (c) F15

	0	1
0	45	2
1	1	24

**Table:** (d) F20

	0	1
0	45	2
1	2	23

**Table:** (e) F25

	0	1
0	45	2
1	1	24

**Table:** (f) F30

	0	1
0	45	2
1	1	24

**Table 14:** Performance analysis of K-FIS using tansig kernel (*γ* = 0.5, *c* = 0.1) with different set of features with best suitable cluster radius (*r*_*a*_ in small bracket).

Models (*r*_*a*_)	Accuracy	Precision	Recall	Specificity	*F*-measure
F5 (0.2)	0.9861	1.0000	0.9615	1.0000	0.9804
F10 (0.2)	0.9722	0.9600	0.9600	0.9787	0.9600
F15 (0.2)	0.9583	0.9600	0.9231	0.9783	0.9412
F20 (0.2)	0.9444	0.9200	0.9200	0.9575	0.9200
F25 (0.2)	0.9683	0.9600	0.9231	0.9783	0.9412
F30 (0.2)	0.9683	0.9600	0.9231	0.9783	0.9412

**Table 15:** Average training, average testing accuracy, and CPU time (in seconds) with different models.

Models∖number of Features	F5		F10		F15		F20		F25		F30	
	Train Acc.	Test Acc.	Train Acc.	Test Acc.	Train Acc.	Test Acc.	Train Acc.	Test Acc.	Train Acc.	Test Acc.	Train Acc.	Test Acc.
KFIS (linear kernel)	95.71	93.06 (2.9)	97.81	97.22 (7.6)	96.86	91.66 (14.7)	94.5	94.44 (24.6)	96.88	95.83 (30.4)	95.98	95.83 (37.1)
KFIS (poly kernel)	98.55	98.61 (44.3)	97.19	97.22 (52.1)	97.83	95.83 (60.2)	94.31	95.83 (79.5)	96.79	95.83 (81.5)	96.76	95.83 (80.7)
KFIS (RBF kernel)	99.24	97.22 (5.5)	95.71	95.83 (13.4)	96.55	97.22 (18.8)	92.12	93.05 (26.1)	92.07	93.05 (31.3)	89.36	87.50 (36.8)
KFIS (tansig kernel)	98.71	98.61 (41.7)	97.19	97.22 (53.4)	97.5	95.83 (69.9)	93.88	94.44 (81.1)	96.92	96.83 (80.7)	96.62	96.83 (84.2)

SVM (linear Kernel)	97.22	97.22 (3)	97.37	97.22 (3.5)	96.61	94.44 (3.6)	97.22	95.83 (3.8)	97.22	95.83 (4)	97.84	97.22 (4.2)
SVM (poly Kernel)	96.75	91.67 (1.5)	96.14	94.44 (1.6)	96.76	93.06 (1.7)	95.83	93.06 (2)	97.22	97.22 (2.2)	97.22	97.22 (2.3)
SVM (RBF Kernel)	97.68	94.44 (2)	97.84	97.22 (2.3)	99.38	100.00 (2.7)	98.00	95.83 (3.2)	98.61	98.61 (3.7)	98.15	98.61 (4.7)
SVM (tansig Kernel)	98.00	97.22 (3.1)	98.30	98.61 (3.3)	98.15	95.83 (3.5)	97.69	94.44 (3.7)	97.22	95.83 (4)	97.84	97.22 (4.7)

## Appendix

For more details see Figures 9, 10, 11, 12, 13, 14, 15, and 16.
